# Affective Game Planning for Health Applications: Quantitative Extension of Gerontoludic Design Based on the Appraisal Theory of Stress and Coping

**DOI:** 10.2196/13303

**Published:** 2019-06-06

**Authors:** Najmeh Khalili-Mahani, Bob De Schutter

**Affiliations:** 1 PERFORM Centre Concordia University Montreal, QC Canada; 2 McGill Centre for Integrative Neuroscience Montreal Neurological Institute McGill University Montreal, QC Canada; 3 Department of Design and Computation Arts Concordia University Montreal, QC Canada; 4 Armstrong Institute for Interactive Media Studies Miami University Oxford, OH United States

**Keywords:** games, user acceptance of health care, psychology, informatics, aging, adaptation, rehabilitation

## Abstract

User retention is the first challenge in introducing any information and communication technologies (ICT) for health applications, particularly for seniors who are increasingly targeted as beneficiaries of such technologies. Interaction with digital technologies may be too stressful to older adults to guarantee their adoption in their routine selfcare. The second challenge, which also relates to adoption, is to supply empirical evidence that support the expectations of their beneficial outcomes. To address the first challenge, persuasive technologies such as serious games (SGs) are increasingly promoted as ludic approaches to deliver assistive care to older adults. However, there are no standards yet to assess the efficacy of different genres of games across populations, or compare and contrast variations in health outcomes arising from user interface design and user experience. For the past 3 decades, research has focused either on qualitative assessment of the appeal of digital games for seniors (by game designers) or on the quantitative evaluation of their clinical efficacy (by clinical researchers). The consensus is that interindividual differences play a key role in whether games can be useful or not for different individuals. Our challenge is to design SGs that retain their users long enough to sustain beneficial transfer effects. We propose to add a neuropsychological experimental framework (based on the appraisal theory of stress and coping) to a Gerontoludic design framework (that emphasizes designing positive and meaningful gaming experience over benefit-centric ones) in order to capture data to guide SG game development. Affective Game Planning for Health Applications (AGPHA) adds a model-driven mixed-methods experimental stage to a user-centered mechanics-dynamics-aesthetics game-design cycle. This intersectoral framework is inspired by latest trends in the fields of neuroimaging and neuroinformatics that grapple with similar challenges related to the psychobiological context of an individual's behaviors. AGPHA aims to bring users, designers, clinicians, and researchers together to generate a common data repository that consists of 4 components to define, design, evaluate, and document SGs. By unifying efforts under a standard approach, we will accelerate innovations in persuasive and efficacious ICTs for the aging population.

## Introduction

### Background

The concept of *healthy aging*, the focus of the World Health Organization’s work on aging between 2015 and 2030, has sparked a growing number of cross-disciplinary research and creation initiatives to take action across multiple sectors to develop strategies for enhancing the autonomy and the well-being of older populations. Among the solutions are the assistive and rehabilitation technologies for the aging population promoted under digital health strategies for remote screening, preventive interventions, or dyadic or machine-coached rehabilitation [1]. Yet, the acceptability and accessibility of these technologies remains a challenge that needs to be addressed, with attention to the complexity of social, economic, and individual contexts of the living experience of older adults [2].

In this position paper, we focus on the gamification of digital training and rehab activities (known as serious games [SG]), which aims to deliver cognitive, emotional, and physical enhancement or rehabilitation routines for older adults [3,4]. For any behavioral intervention to become effective, it must first be acceptable and adaptable to fit the daily routines of the individuals. However, for many older adults, especially those with lower technological literacy or reduced cognitive and physical abilities, the barrier of technological access may be too stressful to overcome. We introduce Affective Game Planning for Health Applications (AGPHA) as an experimental framework based on the appraisal theory of stress and coping to inform SG design and evaluation processes.

Affective game planning builds on the premise that gamification offers a simulated challenge and a corresponding reward system that will motivate users to engage in enjoyable and meaningful health-related activities via information and communication technologies (ICTs). Here, we (1) propose a unifying framework based on a neurobehavioral model of stress and adaptation to address the need for a generalizable empirical approach that accounts for physical and psychological differences in appraisal and adoption of SGs, (2) explain how this model ties to existing theoretical and practical approaches to SG design (namely self-determination and flow theories), with particular emphasis on the necessity of designing enjoyable computerized health applications, and (3) propose a data collection and documentation approach that will enable the field to accelerate research and development of effective and adaptive SGs across multiple applications.

The motivation for this proposal comes from the field of behavioral neuroimaging and methods that it uses to evaluate and document the emotional and cognitive correlates of physiological and environmental phenomena. An influential theory that could explain the psychobiological variations in adaptation and learning, is the appraisal theory of stress by Folkman and Lazarus [5]. In this paper, we propose that the appraisal theory can incorporate 2 dominant game-design theories, self-determination [6] and flow [7,8] and propose a user-centered approach that emphasizes ludic and meaningful experiences in the mechanics-dynamics-aesthetics (MDA) design cycle [9]. We aim to address 2 of the major shortcomings in the field of SG studies: accounting for the complexity of a user’s game-playing experience and preferences [10]; and creating a standard empirical framework to assess the accessibility and efficacy of games that can benefit seniors’ health [11,12].

### History of Games for Health

SG is not a new concept, and promoting the use of digital technologies for improving the quality of cognitive and emotional wellness of older adults has a long history. In their 1976 report of computer use for elder program, Jaycox and Hicks remarked the potential of *game playing* as a use case for building an intergenerational bridge to utilize the new informatics technology for generating easily sharable hypertexts that older generations can use to share experience and knowledge with younger generations [13]. In 1983, Weisman reported the applicability of computer playing in the daily routines of frail elderly (including those with Parkinson disease, dementia, and visual impairment and an average age of 85 years) and showed that 50 of the house residents agreed to repeat play slower adapted versions of 4 games with different skill requirements such as hand-eye coordination (*Brick Out* and *Country Drive*), audiovisual processing and reaction time (*Ribbet*), and memory (*Hangman*) [14]. They remarked the potential for games to increase attention and interest in the players on the one hand and to assist as diagnostic instruments on the other [14]. In 1984, McGuire [15] reported a study to suggest that computer-gaming broke the sedentary routine of residents of nursing homes after he measured elevated moods in 16 adults (compared with 12 nonplaying controls) 8 weeks after they were given access to Atari 2600 games (*Bowling, Football, Breakout, Pac-Man,* and *Space Invaders*). In 1986, Hollander and Plummer [16] introduced 10 games with various cognitive enhancement components (reaction, eye-hand coordination, and memory) in a community house of adults with an average age of 84 years and showed that, in principle, older adults were receptive to the idea of playing games for improving perceptual-motor skills, cognitive abilities, and attention spans from playing various games. The 17 participants who completed 3 weeks of training considered *Trivia* game (questions from popular topics in history, literature, and sports) and a computer-based *Hangman* (a multiplayer computer game to make words with given characters) to be the most interesting of all because they challenged them to think [16]. In 1987, Riddick et al brought 2 upright game arcades to a senior citizens retirement home and introduced them to 2 nonviolent and nonspatial games, *Pac-Man* and *Donkey Kong*, which were slow paced but allowed for progressive skill development—measurable after 19 sessions of playing. They also quantified a significant increase in arousal and recorded the accounts of players explaining why they found playing to be an uplifting experience—despite the fact that the game-play duration and pleasure declined [17].

These historical citations foreground a growing body of similar contemporary work trying to acquire more refined empirical data to support the benefits of more refined digital playing for mental and physical health in older adults.

### Quantitative Serious Game Research

Since a decade, quantitative evaluation of game benefits has returned to the forefront. Basak et al trained 20 seniors (mean age of 69 years) on the personal computer game *Rise of Nations* for a total of 23 hours over the course of 8 weeks and showed improvements in cognitive tests (eg, n-back or mental rotation) compared with the untrained controls [18], even showing evidence for game-related changes in neuroplasticity [19]. Peretz et al performed a randomized trial in 155 seniors and compared a personalized cognitive training protocol versus various computer games assumed to involve cognitive functions (eg, *Tetris*, Target practicing games, and memory games) and showed game-related improvements (albeit weaker than personalized training) in overall cognitive scores [20]. In 2013, an influential publication in the *Nature*, a study of 64 participants (age 65 years, SD 5) provided evidence that training with a multitasking custom game *(Neuroracer)* significantly improved the cognitive control, with effects remaining up to 6 months after the training, concomitant with an increase in the power of theta-band electroencephalogram signals in the medial prefrontal region, an area important for memory and executive control [21]. Toril et al’s meta-analysis of all the literature between 1986 and 2013 revealed that video game training has a positive impact on cognition, albeit the effect size was moderated by factors such as age, personality, and experimental design [22]. However, in a recent (2017) randomized controlled study of *Lumosity* (nonaction cognitive training) versus *The Sims* (active control condition), they found that the control group (*Sims*) became less distracted and faster than the experimental group in performing cognitive tasks [23]. In an independent earlier study (2015), Rose et al demonstrated that simulation of 1-week life planning in the form of a game produced measurable improvements in real-life planning [24]. Overall, it seems that some specific cognitive benefits may be gained from specific elements designed in digital cognition-targeting games [25].

Beside cognitive domains, exercise games (exergames) have been proposed and empirically evaluated for their cognitive benefits, for instance, in older adults with neurological conditions [26], or physical benefits—in terms of improving balance and fall prevention [27-31]. Computer games have also been proposed for cardiac [32,33] and stroke rehabilitation [34]—subject of 3 Cochrane reviews, concluding on the insufficiency of statistical significance because of sampling heterogeneity and inconsistent reporting [35]. Beside quantifiable measures (cognition and physical fitness), which are still debatable [11,36], exercise games provide enjoyment and social interaction [37-44].

### Primacy of Enjoyment in Seniors' Gaming

A recurring theme in studies that target older adults is that *enjoyment* trumps many challenges that would keep them from playing. A text analysis of the content of the literature concerning the older adult’s game experience revealed that many items were related to the potential health benefits of playing digital games, with the top 10 of most recurring terms including *training*, *balance*, *physical*, *cognitive*, *exercise*, and *social* [9]. Despite the general assumptions about technological gaps, older adults, at least in Canada, are onboard for digital play [3]. A recent survey of over 880 older adults in Canada indicates that 73% of the respondents enjoy playing games because they provide social and cognitive stimulation [45]. In a massive online multiplayer game study (*World of Warcraft*), Zhang and Kaufman [46] demonstrated that interindividual variations in enjoyment of the social game experience remain an important determinant of benefiting from the game play experience. Ruvio et al have shown that the *fun factor* in video exercise games predicts adherence to exercise routine compared with the no-game condition [32]. Of course, enjoyment is not the only factor in determining the meaningfulness of a media-related experience [47,48], but in the context of older adults playing, it seems to be important [49]. Many argue that to provide pleasurable and enjoyable experience is the sufficient reason why computer gaming is *good* for older adults [3,48-51].

In the *Gerontoludic Manifesto* (2015), De Schutter and Vanden Abeele have argued that to advance the field of gaming for older adults, we must move away from the biomedical model of solving the problem of age-related decline but instead focus on how different types of games provide a positive experience for their players [52]. Subsequently, in *Gerontodulic Design* (2017), it was proposed that in designing games for older players, the initial step must account for game aesthetics (ie, the emotional response to the games) which reflect the personal essence of gameplay experience and that designers must start by creating enjoyable and playful interfaces that provide connectedness and meaning through implementation of the game mechanics (ie, rules, challenges, and rewards). In this framework, game planning involves gathering iterative player-centered empirical data from the game dynamics (ie, the phenomenology of experiencing the gameplay), as its mechanics evolve through aesthetic improvements [9].

A purely biomedical approach to the problem of design is too reductionist to account for the richness of diverse factors that shape the experience of an SG player, yet designers employ various quantitative approaches and rely on aggregating data through meta-analyses to create a formulaic recipe for enjoyable games that have a higher likelihood of uptake and retention by the players [9,50,53-60].

## Theoretical Framework of Affective Game Planning for Health Applications

### Motivation Theories That Inform Design for Seniors’ Games

At the empirical level, *beneficial* game studies typically rely on well-established psychological motivational theories to understand who plays what, why, and how. In game studies, self-determination theory (SDT) of motivation [6] and flow theory [7,8] are dominant.

SDT posits that humans have an inherent need for autonomy and for developing competencies and relationships that will help them fulfill their goals for growth and self-actualization [6]. This theory is one of the most commonly applied in the field of game studies to explain relations between gaming and psychological need satisfaction [61-63]. The theory is also used as a framework to design gamified strategies for promotion of wellness [64]. Age-related variations in intrinsic and extrinsic motivations that underline SDT are important. In a meta-analysis of 11 independent studies, Birk et al aggregated 3041 samples of postgame player experience of need satisfaction (PENS) and intrinsic motivation inventory data and reported complex and age-dependent correlations between competence, relatedness, and intrinsic motivation [60]. Loos applied the SDT in examining the applicability of exercise games in older adults and found that although the exercise game satisfied the needs for autonomy and competence, they would not recommend the game to others; among the objections were the request for improved user interface or environmental background [65]. In fact, the issue of user interface and cognitive conditions in older adults is pertinent, especially because natural or pathological age-related decline in sensory, motor, and cognitive domains are expected to influence competence and need satisfaction [56,66].

The issues of interface and game accessibility are often addressed with the flow theory [7,8], according to which successful games must counterbalance their features along the axes of challenge and difficulty: if the level of challenge is too high and the player abilities are not commensurate, it leads to anxiety and discontinuation; if the level of challenge is lower than the abilities of the player, it leads to boredom and discontinuation. Nacke et al [67] examined the concept of flow in 2 groups of young and older healthy adults engaging in a pen-and-paper game versus a Nintendo puzzle game and tested the hypothesis that higher challenge associated with the Nintendo task would increase the flow and enjoyment of accomplishing the task. Using this method, they reported that although flow for older adults was correlated with challenge, positive affect, and arousal, for younger players, it was only correlated with challenge and that no affective correlations occurred in the younger sample. Belchior et al used the flow concept to compare the engagement of 45 older adults while training on a laboratory cognitive training stimulus versus training on commercial video games and reported significant increase in flow scores after 6 weeks of playing *Tetris*, as opposed to initially high but diminishing flow after 6 weeks of training on the laboratory game [68]. Marston et al showed different levels of flow experience in older adults trying different exergames, depending on which country the participants were recruited from [69].

### Theory of Appraisal and Coping With Stress

Although SDT underlines the reflective component of playing motivation (competence, relatedness, and autonomy), the flow theory underlines the reflexive aspect (arousal, pleasure, frustration, and success). Our proposed theoretical framework unites these 2 by drawing on Selye’s General Adaptation Syndrome (GAS) [70] and Lazarus’ theory of coping, which incorporate motivational, relational, and cognitive components that give rise to individual differences in perception of, and coping with, stress, which is a physiological phenomenon linked to individual differences in biological factors such as metabolism, immune system, and adaptive learning (for an ontology by Lazarus, see [71]). This theoretical framework is supported by a wealth of evidence that enables us to investigate the interaction between biological and psychological factors that are known to impact not only the momentary experience of the game but also its learning and its long-term impact on broad range of health factors.

#### Biological Manifestation of Stress

Emotional experiences manifest immediate and quantifiable variations in physiological states [72] and cause reflexive embodied experiences (change in heart and breathing rate, galvanic skin response, pupil dilation, facial reflex, movement reflex, and gut reflex). In 1962, to illustrate the power of appraisal on triggering an affective physiological response, Lazarus used 2 silent films, 1 with emotionally charged content and 1 without, and showed significant autonomic responses to the emotive film in terms of heart rate and skin conductance [73]. A wealth of evidence has since accumulated to illustrate the role of this physiological response in enhancing arousal and preparation of the system for an initial *fight-or-flight* response (when the threat is immediate) or a latent *stress* response (when the immediacy of the threat has passed or the threat is of psychological nature) that impacts how one learns from *stressful* experiences [74-77].

In the context of the GAS, we define *stress* as a quantifiable physiological response to any anticipated or actual challenge, intrinsic or extrinsic, real or imaginary, threatening or exciting, that would require an organism to initiate an immediate autonomic response (in the alert/arousal phase) to meet the metabolic demands of extra physical or psychological efforts needed to bring the system back to its normal homeostasis (in the recovery phase) [78]. This physiological response is expected to be different between individuals and represents the sum total of increase on metabolic resources of the body to restore it to baseline, whether they are altered with actual illness or with distressing or joyful perceptions of external stimuli [79].

It is well known that prolonged exposure to stress chemicals can increase the risk of deleterious effects on several body organs, thus negatively affecting the healthy aging process [80]. However, acute stress is not an all-bad response [81] but an important factor for facilitating contextual learning [82,83], for example, through interactions with the reward-processing brain regions [84] or by shifting attention and focus depending on adaptive strategies of individuals [85] (thus, to develop slightly challenging games may serve as a cognition-enhancing activity).

#### Psychological Moderators of Stress

To reify Selye’s biological reductionism, Lazarus proposed the transactional theory of stress and coping, postulating that appraisal and personality could enable one to use cognitive reasoning (recently referred to as mindfulness) to turn a bad stress response (distress and anxiety) to a good experience (eustress, learning, and acting) [5,71]. Lazarus and Folkman have long argued that differences in appraisal and coping strategies influence the dynamics of the GAS [86], and over 50 years of publications in the journal of Psychoneuroendocrinology are dedicated to providing empirical data to illustrate these differences. In general, novelty, unpredictability, and uncontrollability are stressful [87]. A meta-analytical study of over 200 experimental studies by Dickerson and Kemeney [88] provides compelling evidence that stress response is triggered upon perception of threat to the goal of self-preservation (both physical and social self). For instance, physical health is a self-preservation goal. If this goal is threatened by illness or expected surgery, then a stress response will follow. Similarly, if individuals are motivated to preserve their social self by keeping social status, esteem, and acceptance, then they will elicit a stress response (eg, to an exam or public speaking event which challenge this self-preservation goal).

Folkman and Lazarus [86] have suggested that when confronted with a challenging encounter, the primary appraisal process is to categorize it as irrelevant, benign-positive, or *stressful* depending on what implications it would have for their well-being. If the person has no investment in the outcome of the challenge, then they will have no need for it and will not commit to engaging with it. On the contrary, if they perceive immediate or potential benefits, they will experience positive affect. However, if they are not certain about this positive outcome (benign state), then they will enter the secondary appraisal stage, where the individual 'must do something about the challenge.' Therefore, the secondary appraisal focuses on the challenge: 'Is it feasible and within physical and cognitive abilities of the individual or not?'

The appraisal theory of stress postulates that primary and secondary appraisals of a novel challenge with respect to an individual’s actual and perceived emotional and cognitive capabilities would modulate their motivation to approach (learn and play) or avoid the challenge.

As several studies of the playing preferences and patterns in older adults have shown (and we listed them above), they do not consider games to be threatening; however, they do evaluate the impact of games on their wellness by assessing them against their needs and projected benefits [3,48,50,53].

As SGs imply benefits (eg, improving cognitive, emotional, or physical health), then, in the primary appraisal process, they are not irrelevant but positive or benign. This is where the theory of appraisal overlaps the SDT in terms of challenging the user to satisfy their need for competence (Can they learn it?), autonomy (Do they want to learn it?), and connectedness (Does it link them to a greater community?). Next, if the individual decides to play, then the flow theory will apply, as they will ask questions such as: Is the challenge rewarding, arousing, and fun?

If the game is enjoyable and the challenge is not too difficult, then the user will experience flow, the appraisal becomes positive, and the user will keep playing until the self-determination and flow conditions are met. However, if it is too difficult, then the appraisal moves from positive to benign or even *stressful*, running against the individual’s intrinsic need for self-determination and competence. In this secondary appraisal, personality and resources available to the individual will determine the coping process, broadly defined as (1) the ability to deal with functional demands, (2) creating motivation to meet those demands, and (3) maintaining a state of equilibrium that would allow to transfer skills and energy toward those demands [86].

It is at the stage of coping with the challenges of a novel digital gaming experience that the individual will determine whether to turn the *stress* of the challenge into increased attention and practice or to give up and avoid the game stress after a few frustrating tries. According to Lazarus [86], this initial response adjustment is essential in forming later adaptive behaviors in relation to a given challenge. This is where the appraisal theory overlaps the flow theory in terms of arousal, pleasure, and frustration.

#### Neural Correlates of Appraisal in Testing a Game-Like Stressful Stimulus

**Figure 1:**
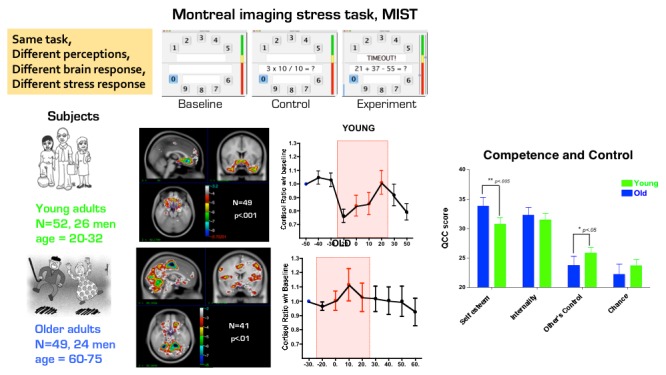
The summary of results from a neuroimaging experiment using a game-like task (MIST). We compared effects of doing metal arithmetics under time- and observer-pressure on brain and stress response in 2 groups of healthy young and older adults. Group differences in personality (Questionnaire of Competence and Control), brain activation (hemodynamic response to experimental versus control), and stress (cortisol) emerged, potentially because of differences in perception of the stressful nature of the task.

As said before, stress is both a biological and a psychological phenomenon and differences in the appraisal of a challenge, together with behavioral coping traits, determine how one approaches or avoids them. Neuroimaging studies of reward, attention, and stress processing can help test transactional models of how a game will exert an immediate or long-term effect on the brain and the subsequent behavioral outcomes. An exhaustive review of this field is beyond the scope of this work, but we draw attention to one of our own observations of how intrinsic differences in perception or differences in cognitive reserves can manifest as distinguishable neuronal activation patterns. In a laboratory stress-simulation study in young healthy adults, we observed that individual differences in physiological stress response to a cognitive stimulus resulted in differences in activation of brain areas involved in learning and emotional processing [89]. We investigated this question in 2 age groups using a game-like mental arithmetic task [90]. This task involved performing simple arithmetic under time pressure, with a competitive element that implied competence if players maintained their performance above the average level. The task became increasingly more difficult if the players’ scores reached the average—without their knowledge. The results are summarized in Figure 1. We observed that when compared with a control condition (performing mathematics without time pressure and without scoring), this task induced different patterns of stress response (measured from hormonal levels and brain activity) in the old and the young participants (with the young being significantly more stressed than the older participants) [91]. Although the young participants engaged the frontotemporal parts of the default mode network (involved in emotional control), the older participants engaged the parietooccipital parts (involved in executive function). This difference was also significant at the level of personality scores—with the young population having lower self-esteem and higher perception of lack of control. This example suggests that age-related differences in perception of the competition and competence or differences in cognitive and emotional reserves may have been linked to significant neurobiological differences.

Hypothetically, if this task was presented as an SG designed for activation of the prefrontal brain regions, then it would not be as effective on the older groups as it would be on the younger ones. Conversely, if this task was administered to increase activation of the posterior parietal regions, then it would be more effective in older adults than the younger ones. In other words, the transfer effects of gamified interventions would significantly vary depending on how players engaged with the game depending on appraisal and affective reaction to the game.

## Experimental Framework of Affective Game Planning for Health Applications

### Quantitative and Qualitative Outcomes

To provide empirical evidence for potential cognitive or health-related benefits appears to be a source of motivation for older adults to adopt computer games in their lives [52,92-95]. Empirical data are also necessary to make recommendations about which serious game to play, and how often [96]. Empirical methods to evaluate game benefits are often focused on evaluated on the expected outcome. In a 2012 review of SGs, Wiemeyer and Kliem [12] have listed a range of physiological functions that can be targeted with SGs, such as cardiovascular and cardiorespiratory system (eg, endurance, cardiovascular fitness, and prevention of cardiovascular diseases), energy metabolism (eg, weight control and prevention of obesity and diabetes mellitus), strength and flexibility (eg, posture and range of motion), bone structure (eg, prevention of osteoporosis), immune system (eg, prevention of cancer), and sensory **–**motor coordination (eg, reaction, balance, and fall prevention). Regardless of the outcome, the game interface mediates the perceptual experiences of activities that target a biological outcome; therefore, gaming is a primarily neuropsychological phenomenon. In other words, the game interface sits between the user’s perception of and interactions with the game and its transferable health outcomes. Although many scientists acknowledge the potential for health SGs, they often neglect the primacy of emotional variables (such as stress, flow or aesthetic experience) in game-related studies of older adults. These issues are often tackled by human-computer-interface (HCI) designers, who emphasize consideration of user’s cognitive and physical abilities that determine their experience and help them overcome challenges related to motivation and preferences [10,97], cultural contexts [53,98], affordance, control and self-determination [54,55,99-101], and accessibility [56,66]. We briefly propose two models that can be tested using a mixed methods approach.

### Biological Model

**Figure 2:**
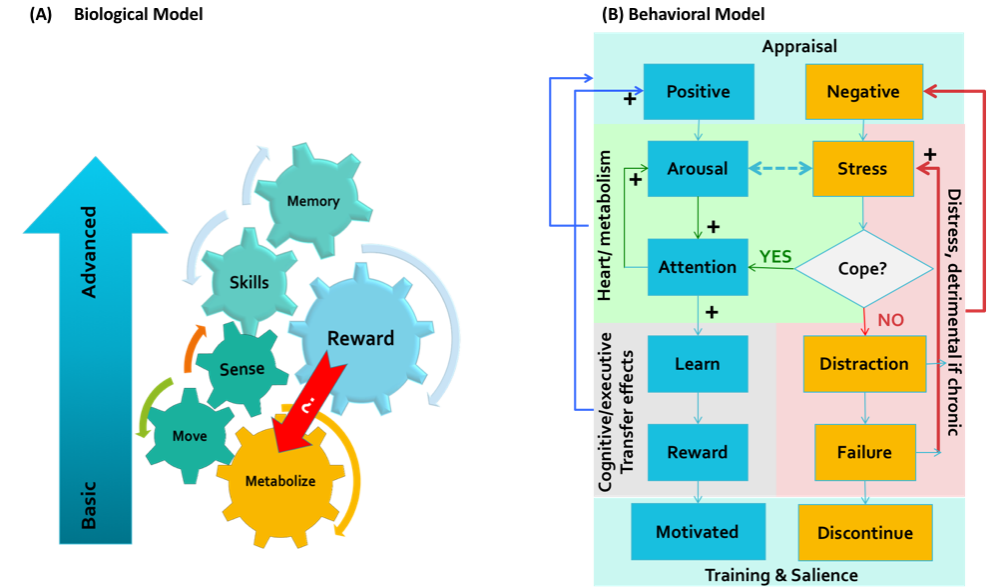
A schematic model of how games can be evaluated based on (a) a biological model (b) or an experimental psychological model of game appraisal to predict its long-term effects.

Consider the model in Figure 2 which illustrates a simplified model of the interrelations between the most basic elements of adaptation. Organisms, from the most basic to the most complex, evolve through an intricate chain of interactions that are tied to basic metabolic regulation of the internal milieu while surviving through a highly variable external milieu (the very bottom of Maslow’s hierarchy of needs) [102]. Brain development involves a chain of interactions with the external world. They begin with sensory processing of the outside information that enters the body through the primary senses: vision, hearing and touch; and movement. Senses and movement further evolve as we grow (or decline as we age) by learning skills that support this process [103]. The genetic and neurochemical signaling pathways that support this learning process are tied to reward and fear processing, and the brain learns the world through an iterative evaluation of the situation and stimuli that are rewarding and enhance its well-being and avoiding or fighting those that threaten it [104-107]. These mechanisms create a closed-loop control system, any of which if broken will put the body in the state of allostatic load (or stress) [108,109]. The physiological manifestation of a stress response reflects the end product of myriad adaptive changes resulting from the interactions between the brain and other organs aimed at restoring the allostasis (ie, the ability to maintain stability through change). Neuroscientists argue that higher human functions, such as communication, creativity, empathy, and playfulness, are all manifestations of the humans extending this exploratory and interconnected terrain—sensing, moving, being rewarded, and learning [110,111]. Whether a game-playing experience produces a sum total of rewarding experiences or not will impact all elements of the system. Individual differences in movement, sensory processing, skills, and learning abilities will determine to what extent an activity is rewarding or stressful as well. We argue that in designing any SG for assistive health care, this big biological picture must be accounted for creating optimal flow experience. Considering that GAS affects the metabolic substrates of behavioral adaptation, the theory of appraisal fits the *big picture* by bridging between behavioral and biological factors that interact during a game-playing experience. For example, one might ask whether appraisal of the game reward will modify behavioral factors such as speed of execution of the game, and metabolic factors such as heart rate, and whether that will transfer to change in higher functions such as hedonic experiences, learning and memory.

### Behavioral Model

Now consider another experimental model in Figure 2, a testable model of how game appraisal will predict later game-playing experience and adoption. The primary point of encounter between a user and a conceptual game is *appraisal* of the value of the exercise. If the game is solely presented as a source of entertainment, then it will have no stressful component, and the choice to play or not will depend on the player’s personal and cultural preferences for pastime. However, if the game is presented in the context of mental or physical health or as a preventive lifestyle strategy, then it implies benefits. At the first encounter with this game, the user will either believe the promise of benefits or reject it as useless or impossible to do. If it is rejected, then they will not further engage in the process. However, if they do subscribe to the beneficial narrative, then the ability to play or not play will become attached to the notion of one’s *self*, and from there on, the interaction with the game will depend on the individual’s appraisal and coping processes with the physical and mental demands of learning and executing the game successfully. In this case, 2 scenarios can unfold that are described below.

#### Scenario 1: When the Game Becomes a Source of Distress

Having accepted the game as a potentially meaningful and health-beneficial activity, if the player perceives the game positively (eg, enjoyable and meaningful), then the player will feel aroused by the game and will pay attention to the gameplay. Games must be challenging to remain interesting, so the player must be challenged enough to fail as the game becomes more challenging. At this moment, secondary appraisal process will engage: “What should I do with the errors?” If the challenge is of value, then the player will increase attention and persevere to learn (eustress) [6,74-77]. If this perseverance results in improvements, then the gameplay becomes rewarding and the player will continue to play (in the state of flow). However, if increased arousal, attention, and practice do not produce positive results (ie, learning and improvement or meaningful connections to their personality or cultural context), then there is a chance that the player would become frustrated and revert to the first appraisal stage: “Is it impossible?”

#### Scenario 2: When the Game Becomes a Source of Eustress

Having accepted the game as a potentially meaningful and health-beneficial activity, if the player perceives the game negatively (eg, too difficult to learn and execute or meaningless to their interest or aesthetic taste), then the experience will become implicitly threatening to their sense of competence and self-efficacy. The player will construe their inability to understand, relate to, and execute the game as a potential source of health-related disadvantage. At this moment, the secondary appraisal process will engage: “What should I do with the fear of failing?” If the game is arousing (either because it is enjoyable or exciting), then the players will increase attention and persevere to learn it, thus turning the stressful context into a reinforcement for learning (eustress). However, if the game is not enjoyable, then each failure becomes a perpetual punishing stressor, a distraction that will block the player’s ability to learn. If this state of frustration is not overcome, the avoidance adaptive mechanism will kick in and, to protect themselves from added distress, the player will abandon the play and seek alternatives.

## Adding Science to Gerontoludic Design

### Design Steps

**Figure 3:**
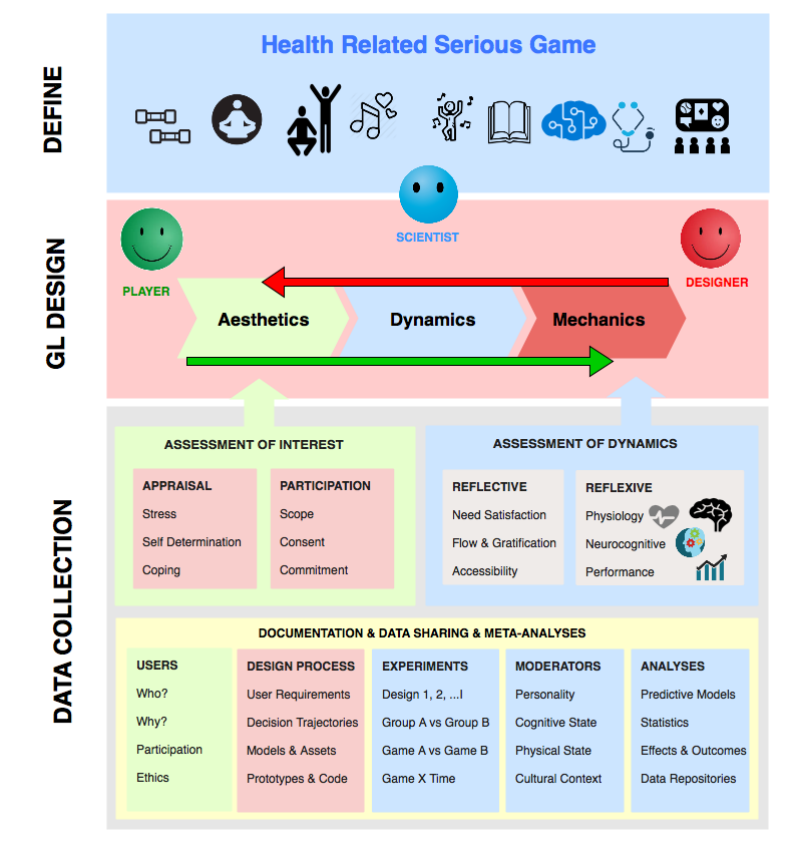
The stages and scope of data collection in the Affective Game Planning for Health Applications framework.

AGPHA offers a scientific framework aimed at the development and evaluation of SGs on a wide range of effectiveness measures. A schematic diagram of the AGPHA is presented in Figure 3. AGPHA is a recursive mixed-methods evidence-based and user-centered process consisting of 4 elements: (1) defining the health-related problem that an SG is to address, (2) identifying or designing a ludic intervention (whether curated from existing games or new), (3) a standardized procedure for data collection during game evaluation or design, and (4) an archiving system that would allow tracking the evolution of the SG design and application over time.

The first step in the process is for scientists to identify the health domain that can be targeted using an interactive ICT, for example, telerehabilitation for stroke recovery, cognitive enhancement, emotional intervention, education, physical fitness, monitoring and data collection, diet and lifestyle, and social networking. As health-related interventions are inherently stressful, the primary aim is to identify or design game-like interfaces that motivate users to learn and sustain playing by minimizing the stressfulness of the activity and maximizing the enjoyment experienced from engaging in the playing.

For this reason, the SGs for health can benefit from a Gerontoludic extension to MDA, which consists of recursive evaluation of the following components:

#### Aesthetics

Typically, the starting point of MDA is for designers to identify emotional outcomes (ie, *aesthetics*) that players will experience as a result of playing a digital game. Considering that the deliverables of AGPHA projects require serious goals or health outcomes, designers and scientists must work together to define general game aesthetics (eg, challenge, fantasy, and competition) in relation to general preferences of older adults (eg, desire for connectedness, meaning, and competence).

#### Dynamics

After defining the initial aesthetics, designers must envision and design a dynamic system that will elicit the game’s intended outcomes. At this stage, design prototypes or existing similar games will be tried to acquire a wide array of subjective and objective game experience measures that can be used to predict whether the game is likely to be enjoyable and effective or too stressful and inaccessible—in which case, the designers go back to reworking the aesthetics.

#### Mechanics

Finally, when a game’s aesthetics and dynamics have been defined, designers can develop the actual mechanics of the game, that is, the rules and components that will elicit the intended dynamics. Again, at this stage, there is a need to evaluate the dynamics of game play and how they change with variations to the rules (eg, speed, challenge, and duration of play). At this stage, the designers and scientists must work closely with users to ensure that both health-related requirements and user requirements for aesthetics and accessibility of the game are met.

### Data Collection

Figure 3 illustrates the scope of data that need to be collected in the AGPHA framework. AGPHA is an iterative mixed-methods approach to user-centered, evidence-based game design. It relies on the evaluation of the reflective (interests) and reflexive (experience) response to games that are developed through the MDA cycle. AGPHA relies on iterative testing and continuous documentation of the process of design and SG evaluation, with a common data thread to enable future aggregate studies.

#### Assessment of Interests

In the AGPHA framework, the first priority is to define the Gerontoludic aesthetics, through appraisal of a conceptual SG, by addressing questions such as: *Does the user believe the promise of the SG benefits, or reject it as useless? If they believe the health promise, then does it fit their value system? Does it make them feel connected? Does it satisfy their need for competence and self-efficacy? Are they willing to try it?* *Do they consent to collecting data from their game playing experience?* The second stage of appraisal begins only if the players have not rejected it right after the first encounter. From then on, the process of interaction with the game will depend on the individual’s secondary appraisal (of the game dynamics) and coping processes. For health-related ICTs to be effective in the older population, they must rely on extensive qualitative studies that evaluate the design aesthetics in the presence of variations in personality and socioeconomic and cultural factors [4,112]. Thus, AGPHA heavily relies on focus groups and on the willingness and consent of the participants to commit to providing multiple quantitative data in the process of co-designing or testing a game. Therefore, experiments in this framework must be designed with regard to ethical considerations of an individual’s privacy, their scope of consent, and the life cycle of the data.

#### Assessment of Dynamics

Game dynamics can be assessed both subjectively and objectively.

The reflective and subjective evaluation of game dynamics can be iteratively recorded using standard questionnaires such as The Player Experience of Need Satisfaction (PENS) that account for various elements of SDT such as competence, autonomy, control, and relatedness [61], or Game Engagement Questionnaire, which measures flow, presence, and immersion [113]), or a more recent aggregated questionnaire that incorporates the two [114]. We also recommend formulating specific questionnaires based on the characteristics of the game’s mechanics (specific skills needed to play the game, content, movement of game elements, and medium on which the game is played), dynamics (game interactions, rewards, speed, flow, and game progression), and aesthetics (how one feels about the game and how it matches their expectations, values, and sensations) [9].

The reflexive and objective response to games can be recoded using physiological signals, which have long served as *biomarkers* of the phenomenological experience of stress in films [115-118] or video games [119]. Measuring physiological responses as a proxy for emotional and hedonic experiences during media consumption has served as a complement to cognitive evaluations or subjective evaluation methods, even in games. In a repeated-measures study of 19 young game players, Poels et al [120] compared 4 different games by examining the predictive value of physiological measures (facial electromyograms and skin conductance as proxies of positive/negative affect and arousal, respectively) in determining the likelihood of repeated and long-term game play. Van Reekum et al [121] measured electrodermal conductivity, forearm electromyograms, and heart interbeat intervals in 33 adolescents playing an action video game and showed an inverse correlation between the magnitude of physiological responses and performance. Mandryk and Atkins [122] used physiological metrics, such as galvanic skin response, facial electromyograms, and cardiovascular responses, and proposed a machine learning algorithm to dynamically compute the degrees of valence and arousal during a game play session in 24 young gamers and showed high convergence between the subjective ratings of games and the machine-predicted levels of emotion and arousal. Hébert et al showed that adding music to the experience of videogame playing caused a moderate increase in the levels of cortisol but no effect on performance metrics [123]. However, to the best of our knowledge, such experiments have not been done in older adults.

One reason for scarcity of such evidence in older adult studies is methodological complexity. Physiological data collection is often a cumbersome activity that requires laboratory protocols to ensure precision and consistency in timing the experiment and recording the experimental events. In addition, the measurement instruments, sensor probes fixed on the surface of the chest, face, or hand skin, are obstructive and extremely sensitive to movement artifacts, making them difficult to apply in a naturalistic gaming experience, especially for older adults. In recent years, neuroimaging methods have also opened an indispensable window into the evaluation of long-term neural benefits or harms of video games [19,21,124-126]. However, these methods are exorbitantly expensive and methodologically demanding, both for researchers and participants; therefore, incorporating such strategies in user-centered design would be impractical.

Recent years have witnessed an exponential growth in the availability of body-worn devices for continuous ambulatory monitoring and reliable wireless data transfer protocols that simplify the experimental setup and reduce the physical and psychological burden on participants. Therefore, opportunities for exploring the relation between reflexive and reflective experience of games in older adults are growing.

Beside physiological signals, several neurocognitive functions, such as short-term memory, reaction time, and attention, are susceptible to stress (eg, Stroop test [127] or short-term memory encode-recall tasks [128,129]). In addition, if the game can be scored easily and consistently, the play scores will supply a quantitative metric of performance. In addition, the long-term transfer effects of games can be measured on different cognitive domains, such as memory, reasoning, or visual speed processing [130].

##### Users

###### Factors in Serious Game Evaluation

Game users cannot be assumed as a monolithic population [10]. Identifying those who do or do not participate in game-design process, or make themselves available to extensive quantitative or longitudinal research, matters. Preferably, anyone who is targeted for SGs must be interviewed to some extent (even if brief) to document their motivation for joining or discarding the research or creation process that is proposed by researchers or designers. The number of individuals who volunteer to participate in study versus those who do not show interest or drop out in the middle is often an important indicator of whether a particular intervention is likely to be useful and adopted. Brox et al [55] and Gerling et al [66] have demonstrated the value of such qualitative data collection over long-term engagement of users in informing the design of senior SGs.

##### Games

Digital games are complex machines that come about through a long, expensive, and iterative decision-making process through collaborations between users, designers, and developers who, in the case of SGs, are guided by the expected health outcome of using the product. Khaled et al have recently outlined the necessity of documentation of the trajectories of the game design process, which revolve around dynamic decision making in confrontation between technological limitations, and human factors [131]. Proper documentation of the software engineering process is equally important, especially in developing health games, where the decision-making process must include expert requirements set by health care professionals [132]. The same factors can be documented when games are selected from existing commercial options (such as Kinect or Wii). Commercial games often have options to customize the interface aesthetics and game mechanics (eg, levels of difficulty) and may provide options to collect performance data.

##### Experiments

Depending on the research question, experimental designs aim to compare different games, different populations, and postgame effects with baseline or longitudinal game effects. However, a common thread running through different experimental designs will make the data amenable to post hoc and meta-analysis [12]. A need for open and standard game play metrics is increasingly acknowledged [133]. The appraisal theory provides a unifying framework that can readily build on a wealth of existing data from the HCI field (accounting for self-determination, flow, use, and gratification and arousal) and generate common data archives from independent but collaborative research by using common metrics and techniques from stress research (See Multimedia Appendix 1).

##### Moderators

The appraisal theory of stress heavily emphasizes the importance of trait factors and interindividual differences in physical and mental coping resources that shape an individual’s approach to a novel challenge (eg, SGs) and predict their physiological and behavioral outcomes of engaging with the challenge over time. Factors such as general self-efficacy, self-esteem, and personality, as well as factors such as socioeconomic status; literacy; mental and physical health; and depression, anxiety or vitality scores, are important variables for which to account—both in statistical analyses and in modeling studies that evaluate the efficacy of games across different experimental conditions—in documents that keep track of the decision-making process in game design or game curation.

##### Analyses

Behavioral studies that account for affective aspects of phenomenological experiences are difficult to replicate, and this is one of the reasons why meta-analytical studies of SG efficacy, even when used to provide physical interventions using exergames, remain inconclusive about which regime and what dose of intervention is more effective [11,12,27]. To remedy this, it is important to clearly document analytical models that are tested on the data and to aim for replicable parsimonious models that provide easily interpretable results about the effect size of a given intervention on an objective and quantifiable metric. Furthermore, physiological data from biosensors are often metricized using postprocessing and data-reduction algorithms, which need to be described to maximize replicability in future studied.

##### Data Sharing and Documentation

To promote SGs for health application in a large scale or to adopt them in clinical rehabilitation routines, researchers must establish their efficacy first. The process of arriving at ideal SG is long, expensive, and often tested in relatively small samples. Given the complexity of cognitive, emotional, and cultural factors that give rise to game aesthetics and the heterogeneity of physical and mental resources that predict the targeted efficacy of game dynamics, it is not possible to make claims about the generalizability of the benefits of SG across different populations (eg, different genders, different ethnicities, and different countries) However, one might hope that adopting a standard framework to trace the evolution of SG development or validation across heterogeneous population or across different aesthetic choices will provide a tremendous opportunity for designing more adaptive and customizable games. AGPHA proposes to ground experimental design based on the appraisal theory of stress and carefully document and consider variations that arise from the following factors:

Future SGs can incorporate data from wearable self-monitoring devices, benefit from ubiquitous computing, and immerse users in customizable aesthetics of virtual and augmented realities. Thus, there is a promising potential for SGs at the heart of persuasive digital health technologies [4]. However, the field is young and in search of methods to establish the clinical relevance and efficacy of these emerging technologies. We draw the attention of health game scholars to research and developments in the field of functional neuroimaging that since 30 years ago have gathered a vast array of techniques and data to examine the link between behavior and neurobiology. Currently, the neuroimaging field has started to concert efforts toward the development of open-source tools and open-data repositories with 2 main objectives: (1) to perform collaborative longitudinal cohort studies to collect large amount of data from different populations using a standardized protocol [134] and (2) to aggregate data from diverse experiments to investigate a common dimension shared by various studies (eg, brain plasticity) [135]. Toward this aim, the AGPHA framework can readily take advantage of several existing platforms such as LORIS [136] and REDCap [137] designed to facilitate data storage for longitudinal and multicenter neurobehavioral studies. These studies have well-established ethical guidelines for lifespan neuroscience studies that may trace an individual in the course of their brain development or aging. Adopting similar standards for data collection, annotation, and documentation ensures adherence to strict policies regarding safety, privacy, and data security of human participants in clinical studies. Such repositories are likely to better inform the design and implementation of SGs based on information gleaned from model-free data mining and machine learning studies on data archives.

## Conclusion and Future Directions

As gamified interventions for health become more *serious*, the need to extend the scope of participatory design to incorporate interdisciplinary, intercultural, and intergenerational concerns grows. Creating empirical and objective frameworks that bring designers, scientists, and technologists together will accelerate development of user-friendly and beneficial digital health strategies for seniors. We have proposed to take advantage of the appraisal theory of stress and coping as a generalizable and unifying framework for evaluation of the efficacy of health SGs. As the appraisal theory encompasses many elements of SDT and flow theory that are prevalent in current game studies, this approach is not a drastic deviation from existing practices. We have shown how this theory can be integrated in the Gerontoludic extension of the MDA design cycle. We also suggest to address the validation requirements of health efficacy of SGs and to draw on resources from neuroimaging and data science to concert efforts in developing adaptive games that address the individual’s needs with regard to their physical, cognitive, and emotional resources.

Ultimately, if the SG research and development community reaches a consensus on how to create longitudinal data repositories in which various aspects of SG research and development can be archived, then we may hope to overcome the problem of small sample sizes and heterogeneity of experimental design that currently limit the scope of proving the clinical effectiveness of SGs. Hopefully, such converging efforts will also lead to developing more persuasive, *intelligent*, and effective SGs that can be tailored to the needs of individuals who need to play them for health benefits.

## Appendix

Multimedia Appendix 1 List of possible instruments that can be used in data collection for Affective Game Planning for Health Applications.

## Abbreviations

AGPHA: Affective Game Planning for Health Applications
GAS: general adaptation syndrome
HCI: human-computer-interface
ICT: information and communication technologies
MDA: mechanics-dynamics-aesthetics
PENS: Player Experience of Need Satisfaction
SDT: Self-Determination Theory
SG: serious games
