# Supplementary material for: Affective Game Planning for Health Applications: Quantitative Extension of Gerontoludic Design Based on the Appraisal Theory of Stress and Coping
Source: JMIR Serious Games. 2019 Jun 6;7(2):e13303. doi: 10.2196/13303 (PMC6592517; doi:10.2196/13303)
Supplement: Multimedia Appendix 1 [file games_v7i2e13303_app1.docx]

**Appendix 1: Requirements of data collection to test the appraisal theory model in AGPHA framework**

In general, AGPHA relies on a cross-over baseline-controlled design that allows repeated measurements of reflexive and reflective data gathered through design iterations, or through comparison of different genres of games. Instruments that can be used in AGPHA framework are listed in Table 1. This list is not exhaustive, but serves to provide a starting point for creation of a common data repository, that will enable us to choose the most discerning tools for assessing interindividual differences in game experience.

1. At baseline, evaluate of the physical and cognitive fitness of all potential users using tests listed in Table 1.
2. A set of control data for post-hoc evaluation of the impact of trait factors, and expectations that can impact the efficacy of SGs
3. Quantitative psychophysiological methods such as those proposed by (1) and employed by HCI researchers such as (2). Inclusion of neuroimaging data acquisition, although expensive and costly, is recommended as it will provide data about the neural substrates of perceptual and executive differences in responding to game, in the short or long term.
4. A live database, such a LORIS (3) that allows researchers to to keep track of individuals’ progress over time, or pool data from similar studies
5. Data-driven and model-driven analytical tools to investigate the dynamics of factors that are measured during the SG evolution process over time (either in the course of design progress, or in in the course of adoption and usage)

Table A1: Instruments that can be used to collect data

| Control Variables |  | Suggested tests |
| --- | --- | --- |
| Cognitive reserve |  | MOCA (4) |
| Physical fitness |  | TUG, 6MWT |
| Affective state |  | State Trait anxiety (19), PANAS (20) |
| Neuropsychology  testing | Attention & Reaction Time | Stroop Color-Word (5) |
|  | Visual Processing | Visual-spatial attention (6) |
|  | Reasoning | Relational processing (7) |
|  | Short term memory | Picture Encode/Recall (8); N-Back (9) |
|  | Reward Processing | Action contingency (10) |
|  | Activity Questionnaires | GetActive (11), Sleep (12) and (13) |
| Game Appraisal |  | Game benefits (14), game attitude (Khalili-Mahani, 2017) |
| Risk Factors |  | Self Efficacy (15), loneliness (16),, PSS (17), vitality (18), General life satisfaction (21) |

**References**

1. 1. Lazarus RS, Speisman JC, Mordkoff AM, Davison LA. A Laboratory Study of Psychological Stress Produced by a Motion-Picture Film. Psychological monographs. 1962;76(34):1-35. PubMed PMID: WOS:A1962CBA5900001.
2. 2. Poels K, Hoogen Wvd, Ijsselsteijn W, de Kort Y. Pleasure to Play, Arousal to Stay: The Effect of Player Emotions on Digital Game Preferences and Playing Time. Cyberpsychology, Behavior, and Social Networking. 2012;15(1):1-6. doi: 10.1089/cyber.2010.0040.
3. 3. Das S, Zijdenbos AP, Harlap J, Vins D, Evans AC. LORIS: a web-based data management system for multi-center studies. Frontiers in neuroinformatics. 2011;5:37. Epub 2012/02/10. doi: 10.3389/fninf.2011.00037. PubMed PMID: 22319489; PubMed Central PMCID: PMCPMC3262165.
4. 4. Nasreddine ZS, Phillips NA, Bedirian V, Charbonneau S, Whitehead V, Collin I, et al. The Montreal Cognitive Assessment, MoCA: a brief screening tool for mild cognitive impairment. Journal of the American Geriatrics Society. 2005;53(4):695-9. Epub 2005/04/09. doi: 10.1111/j.1532-5415.2005.53221.x. PubMed PMID: 15817019.
5. 5. Acevedo EO, Webb HE, Weldy ML, Fabianke EC, Orndorff GR, Starks MA. Cardiorespiratory responses of Hi Fit and Low Fit subjects to mental challenge during exercise. Int J Sports Med. 2006;27(12):1013-22. Epub 2006/04/14. doi: 10.1055/s-2006-923902. PubMed PMID: 16612743.
6. 6. Green CS, Bavelier D. Effect of action video games on the spatial distribution of visuospatial attention. Journal of experimental psychology Human perception and performance. 2006;32(6):1465-78. Epub 2006/12/13. doi: 10.1037/0096-1523.32.6.1465. PubMed PMID: 17154785; PubMed Central PMCID: PMCPMC2896828.
7. 7. Smith R, Keramatian K, Christoff K. Localizing the rostrolateral prefrontal cortex at the individual level. NeuroImage. 2007;36(4):1387-96. Epub 2007/05/29. doi: 10.1016/j.neuroimage.2007.04.032. PubMed PMID: 17532648.
8. 8. Khalili-Mahani N, Dedovic K, Engert V, Pruessner M, Pruessner JC. Hippocampal activation during a cognitive task is associated with subsequent neuroendocrine and cognitive responses to psychological stress. Hippocampus. 2010;20(2):323-34. Epub 2009/05/14. doi: 10.1002/hipo.20623. PubMed PMID: 19437418.
9. 9. Drobyshevsky A, Baumann SB, Schneider W. A rapid fMRI task battery for mapping of visual, motor, cognitive, and emotional function. NeuroImage. 2006;31(2):732-44. Epub 2006/02/21. doi: 10.1016/j.neuroimage.2005.12.016. PubMed PMID: 16488627; PubMed Central PMCID: PMCPMC1620013.
10. 10. Tricomi EM, Delgado MR, Fiez JA. Modulation of caudate activity by action contingency. Neuron. 2004;41(2):281-92. Epub 2004/01/27. PubMed PMID: 14741108.
11. 11. Petrella AFM, Gill DP, Petrella RJ. Evaluation of the Get Active Questionnaire in community-dwelling older adults. Appl Physiol Nutr Metab. 2018;43(6):587-94. Epub 2018/01/18. doi: 10.1139/apnm-2017-0489. PubMed PMID: 29342366.
12. 12. Boeve BF, Molano JR, Ferman TJ, Lin SC, Bieniek K, Tippmann-Peikert M, et al. Validation of the Mayo Sleep Questionnaire to screen for REM sleep behavior disorder in a community-based sample. Journal of clinical sleep medicine : JCSM : official publication of the American Academy of Sleep Medicine. 2013;9(5):475-80. Epub 2013/05/16. doi: 10.5664/jcsm.2670. PubMed PMID: 23674939; PubMed Central PMCID: PMCPMC3629322.
13. 13. Buysse DJ, Reynolds CF, 3rd, Monk TH, Berman SR, Kupfer DJ. The Pittsburgh Sleep Quality Index: a new instrument for psychiatric practice and research. Psychiatry research. 1989;28(2):193-213. Epub 1989/05/01. PubMed PMID: 2748771.
14. 14. Duplaa E, Kaufman D, Sauve L, Renaud L. A Questionnaire-Based Study on the Perceptions of Canadian Seniors About Cognitive, Social, and Psychological Benefits of Digital Games. Games for health journal. 2017;6(3):171-8. Epub 2017/06/20. doi: 10.1089/g4h.2016.0037. PubMed PMID: 28628387.
15. 15. Schwarzer R, Jerusalem M. Generalized Self-Efficacy scale. Measures in health psychology: A user’s portfolio Causal and control beliefs Windsor, England: NFER-NELSON; 1995. p. 35-7.
16. 16. Russell D, Peplau LA, Ferguson ML. Developing a measure of loneliness. J Pers Assess. 1978;42(3):290-4. Epub 1978/06/01. doi: 10.1207/s15327752jpa4203_11. PubMed PMID: 660402.
17. 17. Hong GR, Kang HK, Oh E, Park Y, Kim H. Reliability and Validity of the Korean Version of the Perceived Stress Scale-10 (K-PSS-10) in Older Adults. Res Gerontol Nurs. 2016;9(1):45-51. Epub 2015/09/15. doi: 10.3928/19404921-20150806-72. PubMed PMID: 26366581.
18. 18. Ryan RM, Frederick C. On energy, personality, and health: subjective vitality as a dynamic reflection of well-being. Journal of personality. 1997;65(3):529-65. Epub 1997/11/05. PubMed PMID: 9327588.
19. 19. Bergua V, Meillon C, Potvin O, Ritchie K, Tzourio C, Bouisson J, et al. Short STAI-Y anxiety scales: validation and normative data for elderly subjects. Aging & mental health. 2016;20(9):987-95. Epub 2015/06/10. doi: 10.1080/13607863.2015.1051511. PubMed PMID: 26055726.
20. 20. Crawford JR, Henry JD. The positive and negative affect schedule (PANAS): construct validity, measurement properties and normative data in a large non-clinical sample. The British journal of clinical psychology. 2004;43(Pt 3):245-65. Epub 2004/08/31. doi: 10.1348/0144665031752934. PubMed PMID: 15333231.
21. 21. Peitsch L, Tyas SL, Menec VH, St John PD. General life satisfaction predicts dementia in community living older adults: a prospective cohort study. International psychogeriatrics / IPA. 2016;28(7):1101-9. Epub 2016/02/13. doi: 10.1017/S1041610215002422. PubMed PMID: 26865088.
